# New Perfluorophtalate Complexes of Platinum(II) With Chemotherapeutic Potential

**DOI:** 10.1155/MBD.1996.117

**Published:** 1996

**Authors:** M. B. de Oliveira, J. Miller, R. E. Banks, L. R. Kelland, C. A. McAuliffe, N. Mahmood, I. J. Rowland

**Affiliations:** 1 Federal University of Paraiba Department of Chemistry/CCEN Campus 1 João Pessoa PB 58059-000 Brazil; 2 Federal University of Minas Gerais Department of Chemistry/ICEx Campus of Pampulha Belo Horizonte MG 31270901 Brazil; 3 Federal University of Paraiba Laboratory of Pharmaceutical Technology, Campus 1 P.O. Box 5009 João Pessoa PB 58051-970 Brazil; 4 University of Manchester Institute of Science and Technology Department of Chemistry P.O.Box 88 Manchester M60 1GD United Kingdom; 5 CRC Centre for Cancer Therapeutics Institute of Cancer Research 15 Cotswold Rd Sutton, Surrey SM2 5NG United Kingdom; 6 Medical Research Council Collaborative Centre 1-3 Burtonhole Lane Mill Hill London London NW7 1AD United Kingdom; 7 Institute of Cancer Research Royal Marsden Hospital Downs Rd Sutton, Surrey SM2 5PT United Kingdom

## Abstract

Two new platinum(II) complexes have been synthesized and their anti-tumour and anti-HIV activities have been evaluated.

The new complexes are: (i) *cis*-tetrafluorophthalate-ammine-morpholine-platinum(II) or MMF3 and (ii) *cis*-tetrafluorophthalate- ammine-piperidine-platinum(II) or MPF4. They were characterized by elemental analysis, IR spectra and ^1^H and ^13^C NMR spectra.

They were tested against five human ovarian carcinoma cell lines, *viz.*, CH1, CH1cisR, A2780, A2780cisR and SKOV-3. They were less active than cis-platin and showed cross-resistance with cis-platin in the CH1cisR and A2780cisR acquired resistance lines.

They were also tested for possible anti-HIV activity using the HIV-I IIIB virus and C8166 cells, but they were inactive compared with AZT.


### NEW PERFLUOROPHTALATE COMPLEXES OF PLATINUM(II) WITH CHEMOTHERAPEUTIC POTENTIAL

M. B. de Oliveira[1,2*], J. Miller[2,3*], R. E. Banks[4], L. R. Kelland[5], C. A. McAuliffe[4], N. Mahmood[6], and I. J. Rowland[7]

[1] Federal University of Paraiba, Department of Chemistry/CCEN, Campus 1, 58059-000, João Pessoa, PB, Brazil
[2] Federal University of Minas Gerais, Department of Chemistry/ICEx, Campus of Pampulha, 31270901.Belo Horizonte, MG, Brazil
[3] Federal University of Paraiba, Laboratory of Pharmaceutical Technology, Campus 1, P.O. Box 5009, 58051-970, João Pessoa, PB, Brazil
[4] University of Manchester Institute of Science and Technology, Department of Chemistry, P.O.Box 88, Manchester M60 1GD, UK
[5] CRC Centre for Cancer Therapeutics, Institute of Cancer Research, 15 Cotswold Rd, Sutton, Surrey SM2 5NG, UK
[6] Medical Research Council Collaborative Centre, 1-3 Burtonhole Lane, Mill Hill, London NW7 1AD, London, UK
[7] Institute of Cancer Research, Royal Marsden Hospital, Downs Rd, Sutton, Surrey SM2 5PT, UK

### Abstract

Two new platinum(II) complexes have been synthesized and their anti-tumour and anti-HIV activities have been evaluated.

The new complexes are: (i) *cis*-tetrafluorophthalate-ammine-morpholine-platinum(II) or MMF3 and (ii) *cis*-tetrafluorophthalate-ammine-piperidine-platinum(II) or MPF4. They were characterized by elemental analysis, IR spectra and $^1H$ and $^{13}C$ NMR spectra.

They were tested against five human ovarian carcinoma cell lines, *viz.*, CH1, CH1cisR, A2780, A2780cisR and SKOV-3. They were less active than cis-platin and showed cross-resistance with cis-platin in the CH1cisR and A2780cisR acquired resistance lines.

They were also tested for possible anti-HIV activity using the HIV-I IIIB virus and C8166 cells, but they were inactive compared with AZT.

### Introduction

The 1969 paper by Rosenberg *et al.*(1) on the anti-tumoral activity of platinum complexes markedly increased the interest in metal-based drugs, evidenced by the 1974 review by Cleare(2).

Cis-platin (cis-diammine-dichloro-platinum(II) or CDDP) has potent anti-tumour effects not only on murine tumours such as L1210 leukemia and sarcoma 180(3), but also on various kinds of human tumours including testicular, ovarian, vaginal cervix, lung, bladder, head and neck tumours.

However, it has serious adverse effects such as nephrotoxicity, nausea and vomiting, which clearly limit the efficacy of the drug(4). Many analogues of cis-platin have since been synthesized and tested, in attempts to develop new complexes which may be less toxic and which may have a different spectrum of activity; including activity against cis-platin resistant lines. One of the first new drugs was cis-diammine-(1,1-cyclobutanedicarboxylate)platinum (II), or carboplatin. It has similar activity to cis-platin, but is less toxic. These new analogues were classed as second generation drugs: three of them are shown in Figure 1.

It has been demonstrated recently that CDDP- resistant tumours such as a subline of murine L1210 leukemia(5) and human cell tumour lines(6,7) show a high cross-resistance to CBDCA in studies carried out *in vitro*.

Platinum derivatives are among the most active agents for the treatment of several types of cancer. Prior to the discovery of cis-platin the cure rate in such cases was only 5-10%. However, with current cis-platin based chemotherapy protocols approximately 80-90% of such patients can expect long-term survival free of disease. Following cis-platin based therapy, 30% of patients with stage III ovarian cancer can expect to live for at least 10 years, whereas without it, the survival rate is only about 10%[8,9].

Figure 1. **Some Second Generation Platinum Anti-Cancer Drugs**

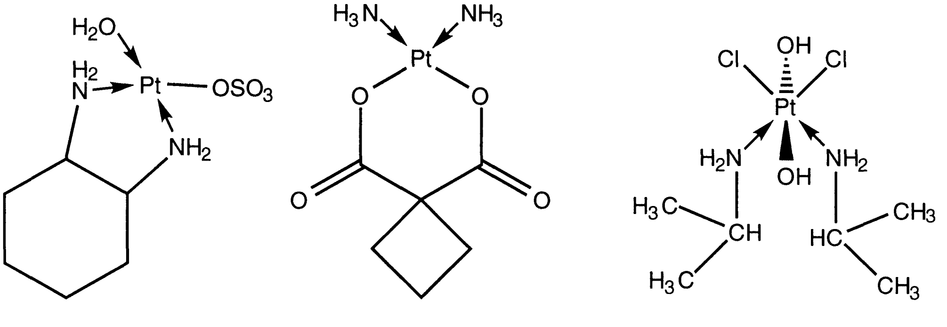

| **Spiroplatin** | **Carboplatin** | **Iproplatin** |
|---|---|---|
| cis 1,2-diamino-cyclohexane aquo-sulphate-platinum(II) | cis-diammine(1,1-cyclobutane-dicarboxylate)platinum(II) | cis-dichloro-bis-isopropyl-amine-dihydroxy-platinum(IV) (CHIP) |

As part of a search for effective platinum-based drugs with lesser collateral effects, differences in hydrophilicity and lipophilicity and in reactivity in biological media, with the potential of oral administration, we have initiated studies with complexes containing organo-fluoro[10] and mixed amine ligands[11].

Perfluoro-carboxylate ligands should be more labile than corresponding complexes without fluorine, just as perfluorocarboxylic acids are more acidic than the corresponding acids without fluorine. However the increase in lability may not be enough to attain that of a chloro ligand. We judge this to be the case for the tetrafluorophthalate ligands. This might lead to longer life in biological media and increase the chances of reaching some tumours. Collateral effects may also be ameliorated.

As regards the amine ligands, changes in these may also modify the intrinsic chemotherapeutic effects as well as altering physical properties and thus distribution of the drug *in vivo*.

With this background, we have prepared two new perfluorocarboxylate complexes containing one ammine and also one amino type ligand. They have been tested for anti-cancer and anti-HIV activity.

### Results and Discussion

The present work is part of a wider study of potential third generation platinum anti-cancer drugs. The selection of the new complexes stemmed from the following considerations:

(i) the complexes should be neutral molecules and with configuration *cis*; (ii) they should contain two different amine ligands; (iii) they should contain a perfluoro-dicarboxylate type ligand.
This may give them lability between those of carboplatin and cis-platin and greater lipophilicity than either. The level of lability in biological media may enable them to reach tumours requiring considerable time for access. They may have greater ability to pass through cell membranes.

It has been suggested for cis-platin that after penetration of the cell membrane[9] the chlorine atoms are displaced by aquo (or possible hydroxy) ligands; while it is the new complex which interacts more readily with the nucleophilic centres of nucleotides.
Figure 2 shows the analogous situation with our complexes.

### Biological Activity

### A) Anti-cancer tests

The tests of compounds MMF3 and MPF4 were carried out at the CRC Centre for Cancer Therapeutics, Institute of Cancer Research, U.K.

**Cell lines**: Five human ovarian carcinoma cell lines were used; three "parent" lines CH1, A2780 and SKOV-3 and two selected for acquired resistance to cis-platin, CH1cisR and A2780cisR. Details of the establishment and biological properties of these lines have been described previously [12]. All lines grew as monolayers in Dulbecco's Modified Eagles' Medium (DMEM) plus 10% heat inactivated foetal calf serum, 50 μg/ml of gentamycin, 2.5 μg/ml of amphotericin B, 2 mM L glutamine, 10 μg/ml insulin and 0.5 μg/ml of hydrocortisone in 10% $CO_2$/90% air. Cells were routinely checked and found to be free of *Mycoplasma.*

Figure 2. **Pictorial view of the new complex being delivered to a tumour cell**

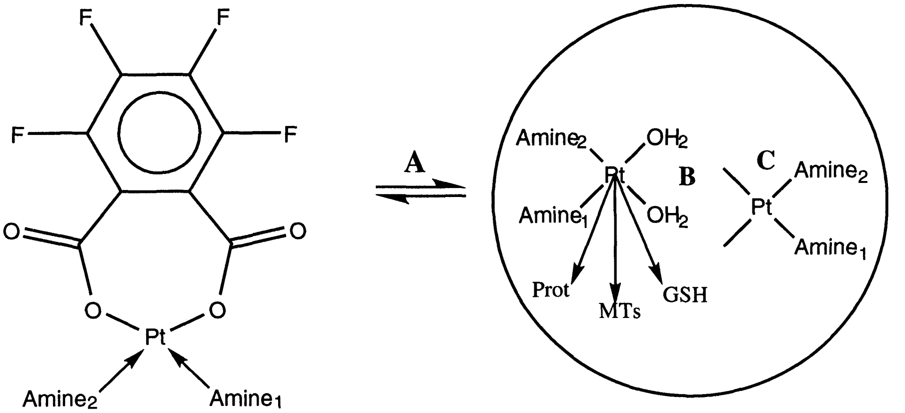

**Assessment of growth inhibition.**

MMF3 and MPF4 were dissolved at 20 mM in DMSO and growth inhibition assessed using the Sulforhodamine B (SRB) assay as described previously[12]. Summarising, cells were seeded into 96-well microtiter plates at 3000-5000/well in 160 μl of growth medium and allowed to attach overnight. Drugs were than added at ten different concentrations (100, 25, 10, 2.5, 1, 0.25, 0.1, 0.025, 0.01 and 0.0025 μM) to quadruplicate wells and left in contact with cells for 96 h. Basic amino acid content per well was then determined by fixing cells in 10% ice-cold trichloroacetic acid, washing with water, staining with sulforhodamine B (0.4% in 1% acetic acid) and solubilising with 100 μl of 10 mM Tris base before determining absorbance values for each well at 540 nm using a plate reader (Titertek multiscan MCC/340). Growth inhibition was then assessed in terms of $IC_{50}$ values (50% inhibitory concentration compared to control untreated cells). Resistance factors ($IC_{50}$ resistant line/parent line) have been determined for the two pairs of cell lines.

**Results**

**Table 1: $IC_{50}$ values (in mM)**

| ovarian carcinoma | MMF3 | MPF4 | Cisplatin |
|---|---|---|---|
| CH1 | 3.4 | 7.3 | 0.1 |
| CH1cisR | 9.5 RF(2.8) | 24 RF(3.3) | 0.7 RF(7) |
| A2780 | 4.95 | 5.2 | 0.3 |
| A2780cisR | 47 RF(9.5) | 42 RF(8.1) | 3.6 RF(12) |
| SKOV-3 | >100 | 50 | 4.4 |

**Conclusions**

Both of the complexes exhibited evidence of *in vitro* anticancer activity against a panel of five human ovarian carcinoma cell lines. In comparison with data obtained under the same experimental conditions for cis-platin, and reported previously[12], the compounds showed a similar pattern of response to cis-platin but with less potency. The $IC_{50}$ values (μM) for cis-platin are included in Table 1. MMF3 and MPF4 generally shared cross-resistance with cis-platin in the two acquired-resistance lines (although resistance factors were a little lower than those obtained for cisplatin) and, as with cis-platin, were not particularly potent against the intrinsically resistant SKOV-3 cell line.

**B) Anti-HIV tests**

The two complexes were tested against the HIV-I IIIB virus and C8166 cells at the Medical Research Council Collaborative Centre, U.K. The results are shown in Table 2 and indicate that the complexes are inactive compared with AZT.

**Table 2: Anti-HIV Activity**

| Compound | Conc (μM) | Syncytia (+/-) | Ag gp120 % of Control | Estimated Cell Growth % of Control | | $EC_{50}$ | $TC_{50}$ |
|---|---|---|---|---|---|---|---|
| | | | | Infected | Uninfected | | |
| MPF4 (2) LH | 50 | + | | 11 | 13 | >20 | 20 |
| | 10 | + | 95 | 15 | 77 | | |
| | 2 | + | 100 | 16 | 98 | | |
| MMF3 LH | 50 | + | | 14 | 44 | >50 | 50 |
| | 10 | + | 86 | 13 | 80 | | |
| | 2 | + | 100 | 17 | 95 | | |
| AZT | 10 | - | | 101 | 101 | 0.016 | >100 |
| | 0.016 | + | 58 | 25 | 100 | 0 | |
| | 0.003 | + | 100 | 24 | | | |
| Control | 0 | + | 100 | 19 | | | |

$EC_{50}$ represents the concentration which reduces the Ag gp120 by 50% in infected cell cultures. $TC_{50}$ represents the concentration of drug which reduces cell growth by 50%.

### Antiviral Assays

The anti-HIV activity and toxicity of compounds were assessed in C8166 cells infected with HIV-I IIIB. The cells were cultured in RPMI 1640 with 10% calf serum. Aliquots of 4 x $10^4$ cells per microtiter plate well were mixed with 5-fold dilutions of compounds prior to addition of 10 $CCLD_{50}$ units of virus and incubated for 5-6 days. The inhibition of infection was measured by examining Synctia and estimating gp 120 antigen in the infected culture supernatant by ELISA, using the lectin GNA (from Galanthus nivalis) to capture the glycoprotein and human anti-HIV serum for detection, as described previously[(13)].

Cell viability of infected and uninfected control cells was measured by the XTF-Formazan method[(14)]. The $EC_{50}$ is the concentration of compound which reduced the production of gp 120 by 50%. The $TC_{50}$ is the concentration of the compound which reduced the viability of uninfected cells by 50%.

### Experimental

### Materials and Methods

IR spectra were obtained using KBr pellets on a Perkin-Elmer 247 spectrometer.

Nuclear Magnetic Resonance spectra were obtained on a Bruker AC 300 spectrometer: $^1H$ spectra were obtained in $CDCl_3$ at 300 MHz and $^{13}C$ spectra in $CDCl_3$ at 75 MHz.

$K_2PtCl_4$ was obtained from Johnson Matthey plc (via UMIST), morpholine and piperidine from the Aldrich Chemical Co and tetrafluorophthalic acid from Fluorochem Ltd.

*Cis*-tetrafluorophthalate-ammine-morpholine-platinum(II) or complex 1 (MMF3) and *cis*-tetrafluorophthalate-ammine-piperidine-platinum (II) or complex 2 (MPF4) are new compounds synthesized as potential third generation drugs.

Their synthesis is outlined in Figure 3.

Figure 3: **Outline Scheme for Synthesis of the Perfluorophthalate Complexes**

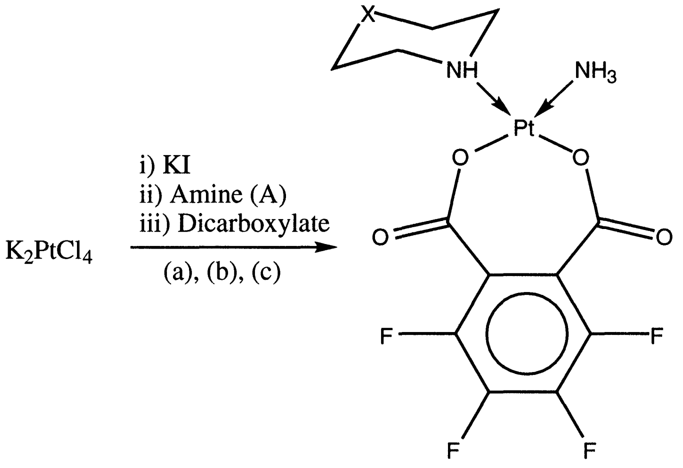

Complex 1; X= O
Complex 2; X= $CH_2$
Amine(A) = Morpholine, piperidine.
Dicarboxylate = Silver tetrafluorophthalate; (a) $H_2O$/EtOH;(b) $HClO_4$ aq./acetone;(c)$NH_3$ aq.

**Complex 1 (MMF3):** 1.0 g of $K_2PtCl_4$ (2.4 mmoles) and 1.6 g of KI (9.6 mmoles) were dissolved in 10 ml of water with stirring. Morpholine (0.42 g; 0.42 ml; 4.8 mmoles) was then added while cooling the reaction mixture in an ice bath. The intermediate (A), *cis*-diiodo-dimorpholine-platinum(II) (*cis*-$[PtI_2(C_4H_9NO)_2]$ precipitated (1.28 g; 2.05 mmoles; 85% yield). It was washed with aqueous ethanol then dried.The intermediate (A) was dissolved in 10 ml of acetone and 2.9 ml of 2.8 M $HClO_4$ aq. solution added with stirring. The precipitate which formed was washed with acetone to give (B) $[PtI_2(C_4H_9NO)]_n$ (1.02 g; 1.1 mmoles; 92% yield). It was dissolved in 10 ml water and 5.7 ml of 1 M $NH_3$ aq. added with stirring. Intermediate (C) (0.93 g; 1.68 mmoles; 88% yield) *cis*-diiodo-ammine-morpholine-platinum(II), precipitated. It was separated, suspended in acetone and 0.3 g (0.64 mmoles) of silver tetrafluorophthalate added with stirring and this continued overnight at room temperature. The mixture was centrifuged, the AgI filtered off and washed with water. The washings and the acetone layer were partially evaporated then combined. The product, MMF3, *cis*-tetrafluorophthalate-ammine-morpholine-platinum(II) was thus obtained in 97% yield (0.85 g; 1.64 mmoles) and recrystallized from acetone.

Elemental analysis (%) calculated for intermediate (C), $[Pt\,I_2\,(C_4H_9NO)(NH_3)]$:
C,8.68; H,2.18; N,5.06; I,45.85; Pt,35.27. Found: C,8.70; H,2.12; N,4.85; I,45.55; Pt,34.80.
Mp 160°C-162°C.
The IR spectrum of intermediate (C) showed bands at $\nu_{max}(cm^{-1})$ 3000-3100(N-H st); 2860(C-H st); 1470(C-H def); 1400-1000 (C-N;C-O-C st); 1260(C-O st); 1200-980 (C-H def); 520 (Pt-N st); 290 (Pt-I st).

Elemental analysis (%) calculated for *cis*-$[Pt\{C_6F_4(CO_2)_2\}(C_4H_9NO)(NH_3)]$, (MMF3):
C,26.93; H,2.26; N,5.23; F,14.20; Pt,36.46. Found: C,25.90; H,2.23, N,4.90; F,13.85; Pt,35.85.
Mp 194°C with decomposition.
The IR spectrum of MMF3 showed bands at $\nu_{max}(cm^{-1})$ 3400-3000 (N-H st); 2860(C-H st of N-$CH_2$); 1640(C=O st); 1470(C-H def); 1400-1100(C-N st); 1360-1140(C-F st); 1260(C-O st); 1200-1000(C-H def); 520(Pt-N st), coherent with the proposed structure.
$^1H$ NMR spectrum (ppm) : Ha 1.7(m); Hb 2.9(m); Hc 3.6(m).
$^{13}C$ NMR spectrum (ppm) : from perfluorophthalate 167(C1-C8); 131(C2-C7 ); 129(C3-C6) and 132(C4-C5). From ammine: 47(C-N) and 70 (C-O).

**Complex 2 (MPF4):** 1.0 g of $K_2PtCl_4$ (2.4 mmoles) and 1.6 g of KI (9.6 mmoles) were dissolved in 10 ml of water with stirring. Piperidine (0.41 g; 0.48 ml; 4.8 mmoles) was then added while cooling the reaction mixture in an ice bath. The intermediate (A), *cis*-diiodo-dipiperidine-platinum(II) (*cis*-$[PtI_2(C_5H_{11}N)_2]$ precipitated (1.38 g; 2.2 mmoles; 92% yield). It was washed with aqueous ethanol then dried.The intermediate (A) was dissolved in 10 ml of acetone and 3.1 ml of 2.8 M $HClO_4$ aq. solution added with stirring. The precipitate was washed with acetone to give (B) $[PtI_2(C_5H_{11}N)]_n$ (1.03 g; 1.9 mmoles; 86%

yield). It was dissolved in 10 ml water and 5.7 ml of 1 M $NH_3$ aq. added with stirring. Intermediate (C) (0.70 g; 1.2 mmoles; 67% yield) *cis*-diiodo-ammine-piperidine-platinum(II), precipitated. It was separated, suspended in acetone and 0.32 g (0.7 mmoles) of silver tetrafluorophthalate added with stirring and this continued overnight at room temperature. The mixture was centrifuged, the AgI filtered off and washed with water. The washings and the acetone layer were partially evaporated then combined. The product, MPF4 *cis*-tetrafluorophthalate-ammine-piperidine-platinum(II) was thus obtained in 66% yield (0.23 g; 0.4 mmoles) and recrystallized from acetone.

Elemental analysis (%) calculated for intermediate (C), *cis*-$[PtI_2(C_5H_{11}N)(NH_3)]$:
C,10.89; H,2.56; N,5.08; I,46.06; Pt,35.40. Found: C,11.03; H,3.12; N,4.85; I,45.85; Pt,36.01.
Mp 160°C with decomposition.
The IR spectrum of intermediate (C) showed bands at $\nu_{max}(cm^{-1})$ 3400-3000(N-H st); 2930-2860(C-H st); 1600 -1470(N-H def.); 1350-1000(C-N,N-H st); 510 (Pt-N st); 300(Pt-I st).

Elemental analysis (%) calculated for *cis*-$[Pt\{C_6F_4(CO_2)_2\}(C_5H_{11}N)(NH_3)]$, (MPF4):
C,29.29; H,2.64; N,5.25; F,14.25; Pt,36.60 Found: C,30.20; H,2.64, N,4.95; F,13.80; Pt,35.98.
Mp 189°C-191°C.
The IR spectrum of $MPF_4$ showed bands at $\nu_{max}(cm^{-1})$ 3300-3000(N-H st); 2860(C-H st); 1630(C=O st); 1470(C-H def); 1400-1100(C-N st); 1340-1140(C-F st); 1200-1000(C-H def); 510(Pt-N st), coherent with the proposed structure.
$^1H$ NMR spectrum (ppm): Ha and Hb, 2.8 (m); Hc 1.6 (m).
$^{13}C$ NMR spectrum (ppm): From tetrafluorophthalate 167(C1-C8); 130(C2-C7); 128(C3-C6) and 131(C4-C5). From ammine: 48(C-N); 34(C*-C-N) and 27(C-C*-C) ppm.

**Acknowledgements**

The authors thank CNPq (Conselho Nacional de Desenvolvimento Científico e Tecnológico) for a Post-doctoral Fellowship (MBO); Johnson Matthey plc for a gift of $K_2PtCl_4$ (via UMIST); Fluorochem Ltd. for tetrafluorophthalic acid and, Dr. C. Peppe of the Organometallics Laboratory of the Federal University of Paraiba for the concession of laboratory facilities.

**References**

(1) Rosenberg, B., van Camp, L., Trosco, J.E and Mansour, V.H., *Nature*, **222**, 385-86 (**1969**)
(2) Cleare, M.J., *Coord. Chem. Rev.*, **12**, 349 (**1974**)
(3) Burchenal, J.H., Kalaher, K., Dew, K. and Lokys, L., *Cancer Treat. Rep.*, **63**, 1493-98 (**1979**)
(4) Connors, T.A., Cleare, M.J. and Harrap, K.R., *Cancer Treat. Rep.*, **63**, 1499-1502 (**1979**)
(5) Kraker, A.J. and Moore, C.W., *Cancer Res.*, **48**, 9-13, (**1988**)
(6) Behrens, B.C., Hamilton, T.C., Masuda, H., Grotzinger, K.R., Whang-Peng, J., Louie, K.G., Knutsen, T., McKoy, W.M., Young, R.C. and Ozols, R.F., *Cancer Res.*, **47**, 414-18 (**1987**)
(7) Koichi, E., Ken-Ichi, A., Tomoko, M., Kazumi, M., Masuo, K., Hiroki, M. and Kinya, K., *Anticancer Res.*, **12**, 49-58, (**1992**)
(8) Neijt, J.P., ten Bokkel Huinink, W.W., van der Burj, M.E.L., van Oosterom, A.T., Willemse, P.H.B., Vermorken, J.B., van Lindert, A.C.M., Heintz, A.P.M., Aartsen, E., van Lent, M.,Trimbos, J.B. and Meijer, A.J., *Eur. J. Cancer*, **27**, 1367 (**1991**)
(9) Kelland, L.R., Clarke, S.J and McKeage, M.J., *Platinum Metals Rev.*, **36**(4), 178-184 (**1992**)
(10) Oliveira, M.B., Doctoral Thesis, University of São Paulo, USP, Brazil, (**1993**)
(11) Hefferman, J.G and Hydes, P.C., *Eur. Pat. Appl.*, 0167310 (**1985**)
(12) Kelland, L. R., Abel, G., McKeage, M.J., Jones, M., Goddard, P.M., Valenti, M., Murrer, B.A. and Harrap, K.R., *Cancer Res.*, **53**, 2581-86 (**1993**)
(13) Mahmood, N. and Hay, A.J., *J. Immunol. Methods.*, **151**, 9 (**1992**)
(14) Weislow, O.S., Kiser, R., Fine, D.L., Bader, J., Snoemaker, R.H., and Boyd, M.R., *J. Natl. Cancer Inst.*, **81**, 577 (**1989**)

**Received: February 22, 1995 - Accepted: March 7, 1995 - Received in revised camera-ready format: March 26, 1996**

